# A Green Lantern for the Surgeon: A Review on the Use of Indocyanine Green (ICG) in Minimally Invasive Surgery

**DOI:** 10.3390/jcm13164895

**Published:** 2024-08-19

**Authors:** Pietro Fransvea, Michelangelo Miccini, Fabio Rondelli, Giuseppe Brisinda, Alessandro Costa, Giovanni Maria Garbarino, Gianluca Costa

**Affiliations:** 1 Emergency Surgery and Trauma, Fondazione Policlinico Universitario “A. Gemelli” IRCCS Roma, Catholic University of Sacred Heart, 00136 Rome, Italy; pietro.fransvea@policlinicogemelli.it (P.F.); giuseppe.brisinda@policlinicogemelli.it (G.B.); 2 Department of Surgery, Sapienza University of Rome, 00185 Rome, Italy; michelangelo.miccini@uniroma1.it; 3 Department of Medicine and Surgery, University of Perugia, 06123 Perugia, Italy; fabio.rondelli@unipg.it; 4 UniCamillus School of Medicine, Saint Camillus International University of Health and Medical Sciences, 00131 Rome, Italy; alessandro.costa.16.10@gmail.com; 5 Department of Surgery, Ospedale Sant’Eugenio, ASL RM2, 00144 Rome, Italy; 6 Department of Life Science, Health, and Health Professions, Link Campus University, 00165 Rome, Italy

**Keywords:** indocyanine green, minimally invasive, surgery

## Abstract

Indocyanine green (ICG) fluorescence imaging has revolutionized surgical practice across various medical and surgical specialties. This article reviews the clinical applications of ICG in abdominal, urological, thoracic, and gynecological surgery. ICG fluorescence imaging has been widely adopted in general surgery for various applications, including perfusion assessment, intraoperative visualization of the ureter, and tumor localization. It is particularly valuable in evaluating anastomotic leaks and aiding in precise tumor resection during minimally invasive surgeries. Studies have shown mixed results on its effectiveness in reducing anastomotic leak rates, highlighting the need for further research. In thoracic surgery, ICG facilitates the identification and resection of pulmonary bullae, as well as the precise localization of pulmonary nodules during video-assisted surgery. In urology, ICG aids in localizing renal tumors and guiding selective arterial occlusion during partial nephrectomy. Its role in identifying the lymphatic pathway in prostate cancer and sentinel lymph node biopsy in gynecological cancer is also discussed. Despite its benefits, the use of ICG fluorescence faces challenges such as limited tissue penetration, the potential for false results, a lack of standardized protocols, and high equipment costs. Nonetheless, it remains a powerful tool that could improve surgical outcomes.

## 1. Introduction

“In the blackness of the night, I see a sparkle of a star” sings the lyrics of a song by Yusuf Islam/Cat Stevens. Indeed, indocyanine green (ICG) proves to be a useful illumination source that aids in the “blackness” of many challenging surgical procedures. One fundamental principle in surgery is “never cut what you can’t see” [1]. However, when visual cues are limited, surgeons rely on other senses for guidance. In minimally invasive procedures, the challenge of working without direct tactile feedback and with two-dimensional vision constantly challenges this principle. Distinguishing between structures that need to be removed, such as tumor tissue and lymph nodes, and those that must be preserved, such as blood vessels and bile ducts, is crucial in everyday surgical scenarios [2,3,4,5]. Kitai’s pioneering work on utilizing ICG for fluorescence-guided surgery (FGS) while performing sentinel node biopsy for breast cancer opened a new era of image-guided surgery in the realm of general surgery [6]. Since then, ICG fluorescence imaging has become an extensive scope of research. In the past, ICG, a water-soluble tricarbocyanine dye, found widespread use in determining cardiac output, evaluating hepatic function and liver blood flow, and investigating chorioretinal disorders. Its safety profile is robust, with adverse events rarely occurring below a dosage of 0.5 mg/kg. Upon intravenous administration, ICG swiftly binds to plasma lipoproteins, exhibiting a plasma half-life of 3–5 min and biliary excretion within 15–20 min. Then, ICG is cleared exclusively through the liver and then eliminated through the bile. It does not undergo metabolism. In the literature, there are no complications associated with the use of ICG for surgery. It is known that in very rare cases, the injection of preparations of indocyanine green can cause nausea and anaphylactoid or anaphylactic reactions (<1/10,000). In patients with terminal renal failure, the possibility of an anaphylactic reaction seems to have increased, and spasms of the coronary artery have also been described.

Regarding the dosage and timing, based on the literature, there is no standard. The timing and dose depend on the type of surgery and for what the fluorescence is used. Moreover, doses are device-dependent. The International Society of Fluorescence Surgery developed a table of ICG dosing for a single type of procedure based on recommendations from worldwide surgeon experts in any single procedure, but this is not based on evidence. Both excitation and fluorescent lights are in the near-infrared region where light absorption by hemoglobin (and water) is small, so the light can travel long distances in the human body. Indocyanine green (ICG) can be excited and emits fluorescence, which can be visualized by appropriate camera systems approved worldwide. Live images from the fluorescent dye and the surgical field are obtained using a combination of filters, lenses, and cameras. During open surgery, hand-held devices are usually preferred for their ease of use and mobility. Guided Surgery (FGS) can also be performed using minimally invasive devices such as laparoscopes or endoscopes. In this case, a system of filters, lenses, and cameras is attached to the end of the probe. There are a variety of camera systems approved and on the market that are used in conjunction with indocyanine green (ICG) dye. Fluorescence-Guided Surgery (FGS) devices can also be implemented for robotic surgery (for example, in the da Vinci Surgical System) [7,8,9,10,11,12]. This facilitates the real-time identification of anatomical structures and the assessment of blood flow. Augmented reality, leveraging ICG’s fluorescence, augments conventional senses, offering precise anatomical localization and real-time tissue functional data. Recently, several applications have been introduced in daily clinical practice, ranging from oncologic and acute abdominal surgery to thoracic surgery, urology, and gynecology [13,14]. Particularly in complicated scenarios marked by inflammatory states and compromised blood supply due to underlying conditions, this technology holds promise as a surgical game-changer [15,16]. We herein present an overview of the principles of ICG imaging and its clinical use in different surgical fields (Table 1).

## 2. Material and Methods

A comprehensive literature search was conducted to identify relevant studies on the use of indocyanine green (ICG) in surgery. The PubMed, Scopus, Web of Science, and Cochrane Library databases were systematically searched from their inception until [30 June 2024]. The following keywords and Medical Subject Headings (MeSH) terms were used in various combinations: “indocyanine green”, “ICG”, “fluorescence imaging”, “near-infrared imaging”, “surgery”, and “intraoperative”. Boolean operators (AND, OR) were applied to refine the search strategy. Articles were included if they focused on the application of ICG in surgical procedures, were peer-reviewed original research articles, clinical trials, case series, or meta-analyses, and were published in English. Exclusion criteria included non-English language publications and studies without a specific focus on the surgical use of ICG. Two independent reviewers screened the titles and abstracts of all identified studies. The full texts of potentially relevant articles were retrieved and assessed for eligibility. Data extracted from the selected studies included study design, patient demographics, surgical procedures, ICG administration protocols, imaging techniques, outcomes measured, and any reported complications or limitations. Discrepancies between reviewers were resolved through discussion, and if necessary, a third reviewer was consulted. Given the narrative nature of this review, a qualitative synthesis of the data was performed. The findings were grouped based on the type of surgery (e.g., oncological, urologic, thoracic, etc.) and the specific role of ICG (e.g., tissue perfusion assessment, lymphatic mapping, tumor margin delineation). The trends, benefits, and limitations of ICG use in different surgical contexts were analyzed and summarized. Regarding ethical considerations, as this study involved a review of previously published data, ethical approval was not required.

## 3. Abdominal Surgery

Currently, several recent applications have been introduced into daily clinical practice. Its uses include perfusion assessment, intraoperative visualization of the ureter, identification of sentinel nodes, and visualization of lymphatic drainage. It has also been used to localize and assess peritoneal and hepatic metastases [16,17,18,19,20,21,22,23,24,25].

### 3.1. ICG Use for Perfusion Assessment

**Figure 1:**
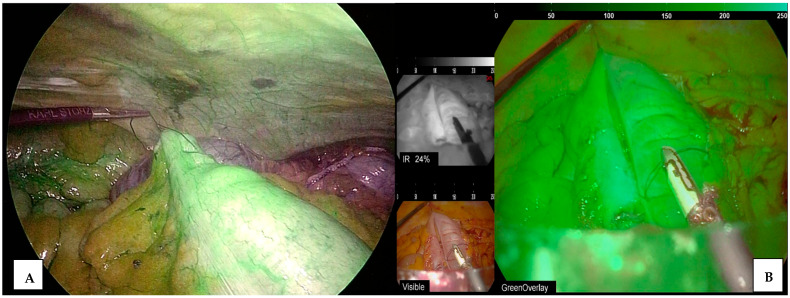
ICG use for perfusion assessment during ileo-colic anastomosis. (**A**) Perfusion control ileocolic anastomosis (colonic side), (**B**) evaluation of enterotomy closure perfusion.

Regarding perfusion assessment, ICG finds valuable utility, particularly in evaluating anastomotic leakage [26,27,28]. The occurrence of anastomotic leaks varies according to the surgical procedure, with rates of this concerning postoperative complications reaching up to 19% [29,30]. In any gastrointestinal surgical procedure involving resection, ensuring proper healing of the anastomosis is crucial, requiring adequate vascularity and tension-free connections (Figure 1). ICG offers a valuable means to intraoperatively evaluate anastomotic perfusion [31,32,33,34,35]. In 2018, Ris et al. conducted a multicenter, prospective, phase II study aiming to evaluate the potential of indocyanine-green-guided fluoresce angiography (ICG-FA) in guiding the selection of intestinal transection levels and assessing subsequent anastomotic vascular sufficiency. This study enrolled 504 patients who underwent colorectal resection for benign and malignant diseases, defining anastomotic leakage (AL) according to Clavien–Dindo (CD) grade ≥ 3. Among them, the planned transection line was adjusted based on ICG-FA findings in 24 patients (4.7%). In five out of ninety patients who underwent low anterior resection, the decision to construct a diverting stoma was reconsidered based on ICG-FA results. Consequently, the surgical approach was modified for 29 patients (5.8%) due to the utilization of ICG-FA. The overall AL rate for colorectal resection in this study was 2.6%, significantly lower than the rate of 5.8% observed in 1173 procedures performed without ICG-FA at the participating centers [36]. In 2019, Dinallo et al. conducted a retrospective study involving 554 cases of colorectal resection, comparing those with and without ICG-FA for benign and malignant diseases. The study found no significant difference in AL rates between the ICG-FA and control groups. However, the transection line was adjusted in 5.6% of cases in the ICG group, which was significantly higher compared to the control group [37]. However, the majority of these studies were single-center investigations, and considerable discrepancies existed in the definition of AL, the specific disease under study, the surgical procedures performed, and various other background factors influencing the occurrence of AL. In 2020, Alekseev et al. presented the findings of the FLAG trial, a randomized controlled trial conducted at a single center in Russia, which involved 377 patients undergoing sigmoid and rectal resection. The results revealed a notable reduction in the rate of anastomotic leakage (AL) in the ICG-FA group compared to the control group, particularly among the 216 patients who underwent low anastomosis [38]. In 2021, Jafari et al. presented findings from the PILLAR-III trial, a multicenter randomized controlled trial conducted across 25 U.S. centers. This trial enrolled 347 patients with rectal neoplasms who underwent low anterior resection with anastomoses located less than 10 cm from the anal verge. The study results did not indicate a significant reduction in the AL rate in the ICG-FA group compared to the control group [39]. Thus, while previous randomized controlled trials did not provide evidence suggesting a significant reduction in AL rates with the use of ICG-FA, recent developments have emerged. The authors of a randomized control trial (RCT) conducted in Japan presented promising findings at the 30th International Congress of Endoscopic Surgery in the European Union in 2022. This study, known as the EssentiAL study, was a multicenter RCT involving 41 Japanese centers analyzing 839 patients who underwent sphincter-preserving surgery for rectal cancer. The results demonstrated a significantly lower AL rate in the ICG-FA group compared to the control group.

### 3.2. ICG Use for Tumor and Sentinel Lymph Node Localization

**Figure 2:**
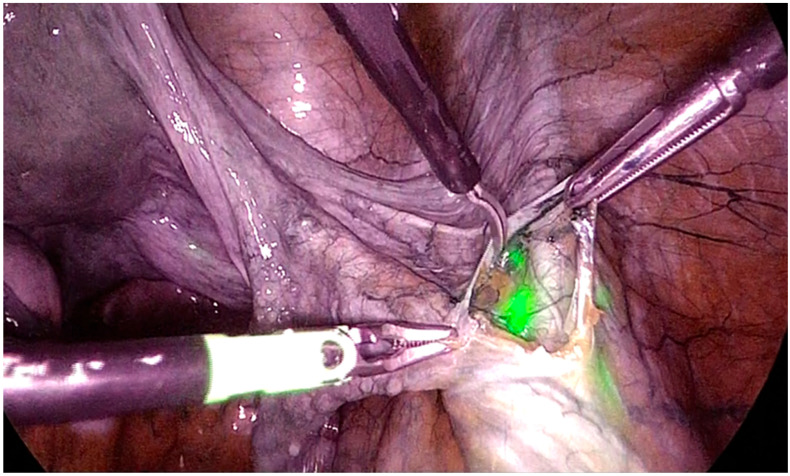
ICG use for iliac sentinel lymph node localization.

Another application of ICG in abdominal surgery is tumor localization [40]. While minimally invasive treatments offer the advantage of reducing postoperative recovery time and enhancing long-term quality of life for patients, the absence of direct tactile feedback during laparoscopic or robotic procedures poses challenges, particularly in the early stages of cancer when infiltration of the gastrointestinal serosa has not occurred [23]. This limitation complicates intraoperative tumor localization and subsequent determination of the resection line. Various methods have been proposed to address this issue, including preoperative submucosal injection of India ink, the application of titanium clips, and direct intraoperative endoscopic observation [41,42,43,44,45,46]. However, these approaches often prolong intraoperative detection time and effort, and there is a risk of ink leakage compromising the surgical field. To circumvent these challenges, ICG emerged as the preferred option due to its enhanced visibility under specific lighting conditions, remaining invisible in natural light and thereby minimizing interference with the surgical field (Figure 2). Moreover, in radical surgeries for gastric cancer and colorectal cancer, there are instances where it becomes necessary to reduce the surgical margin distance to spare normal tissues while ensuring a safe margin distance. Preserving more healthy gastric tissues can significantly enhance patients’ postoperative quality of life. Ultra-low anastomosis in rectal cancer surgery presents a valuable option for patients seeking to retain their anus, with precise tumor localization being key to its successful execution, ensuring the preservation of an adequate length of the lower rectum. The potential benefits of successfully implementing ICG fluorescence imaging (ICG-FI) in improving patients’ long-term quality of life after surgery are evident. Furthermore, some studies have explored the use of preoperatively placed fluorescent clips during endoscopy to aid intraoperative tumor localization via fluorescent signals emitted by the clips [47,48,49,50,51,52]. The initial use of ICG for sentinel lymph node (SLN) detection started with gastric cancer [53,54]. The SLN, being the primary station of lymphatic drainage for gastric cancer, is highly susceptible to metastasis. However, the application of SLN navigation surgery in gastrointestinal tumors remains contentious, with complexities such as micro metastasis and skip metastasis in gastric cancer’s intricate lymphatic drainage. Accurate identification of the SLN remains a key focus for gastrointestinal surgeons. A multicenter prospective trial in Japan employed the endoscopic dual-tracer method, combining radiolabeled tin colloid and blue dye, for SLN detection in clinical stage T1 primary gastric cancer of 4 cm or less, yielding favorable results. The dual-tracer method achieved a high SLN detection rate and accuracy in assessing metastatic status based on SLN evaluation [55]. However, challenges such as invasiveness, restrictions on radioactive colloid use, and high medical costs hinder its clinical applicability. The advantages of ICG, including safety, convenience, affordability, and a shorter learning curve, outweigh those of radioactive tracers. Tajima et al. demonstrated successful SLN identification using ICG in both laparoscopy-assisted gastrectomy and open gastrectomy. In the laparoscopy-assisted gastrectomy group, the accuracy and false-negative rates were high [56].

### 3.3. ICG in Upper-GI Surgery

Regarding upper-GI surgery, despite recent technological innovations and the development of minimally invasive surgery, esophagectomy remains an operation burdened with severe postoperative complications because many factors could affect anastomotic healing. Among the surgical-related factors, the aspects of paramount importance are the tension of anastomosis and its location, the technique, and the length of the conduit affecting vascularization. Although experienced surgeons are able to evaluate the blood flow by de visu inspection, ICG is a possible tool for a more objective evaluation of the gastric conduit. Based on a review by Tamburini N et al., ICG seems to be a promising and safe method for reducing surgical morbidity following esophageal resection with continuity restoration [57]. The authors reported that anastomotic leakage was reduced using ICG fluorescence angiography. This finding was confirmed by the study of et Koyanagi K et al. who reported that, although there was considerable interstudy heterogeneity among the patients, a decrease in the incidence of anastomotic leakage was observed when blood perfusion in the gastric conduit was evaluated objectively by ICG fluorescence imaging. Unfortunately, there is a lot of variability in the way this tool has been used and what the results indicate. Moreover, future studies are needed to establish the feasibility of ICG lymphography to prevent chyle fistulas and guide lymphadenectomy [58].

### 3.4. ICG in Laparoscopic Cholecystectomy

**Figure 3:**
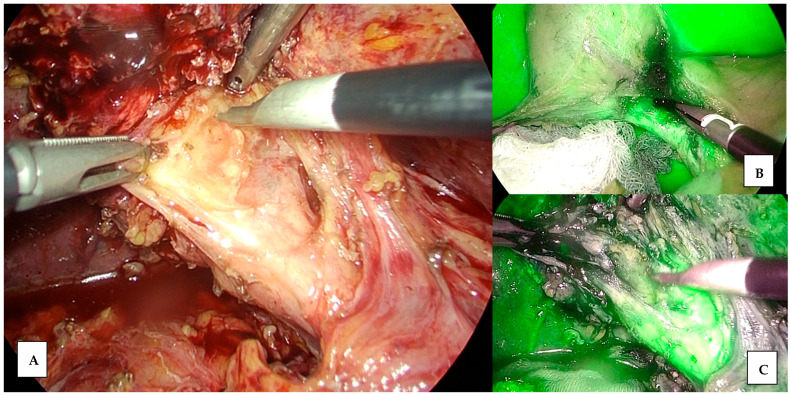
Structure identification during cholecystectomy. (**A**) Difficult identification of Calot triangle with normal view. (**B**) Improvement of identification of Calot’s triangle with ICG. (**C**) Dissection of Calot’s triangle elements with ICG vision.

Another field of application of ICG fluorescence is elective minimally invasive cholecystectomy. Laparoscopic cholecystectomy (LC) remains one of the most commonly performed procedures in adult and pediatric populations. Despite the advances made in intraoperative biliary anatomy recognition, iatrogenic bile duct injuries during LC represent a dramatic complication, sometimes even life-threatening. Moreover, bile duct injuries constitute an economic burden for healthcare systems. Based on the literature, a series of methods have been proposed to prevent bile duct injury, with the use of ICG fluorescence among them (Figure 3). Despite widespread adoption and evaluation in recent years, variations persist in ICG’s clinical utility, including the dose, concentration, and timing of administration. The most commonly reported method of ICG injection is intravenous administration, while the literature lacks studies investigating the direct injection of ICG into the gallbladder [59,60,61]. In a mini review by Symeonidis S. et al. including four prospective cohort studies, three case–control studies, and one case report, intra gallbladder ICG injection is a promising method to achieve biliary mapping, overcoming the limitations of intraoperative cholangiography including intervention and radiation exposure, as well as the high hepatic parenchyma signal and time interval needed in intravenous ICG fluorescence [62]. The same findings were reported by the meta-analysis performed by LIM SH, which compared ICG cholangiography with intraoperative cholangiography in minimal-access cholecystectomy for visualization of the extrahepatic biliary tree. concluding that it is safe and improves visualization of the bile duct [63].

### 3.5. ICG Use in Hepatobiliary Surgery and Transplant

**Figure 4:**
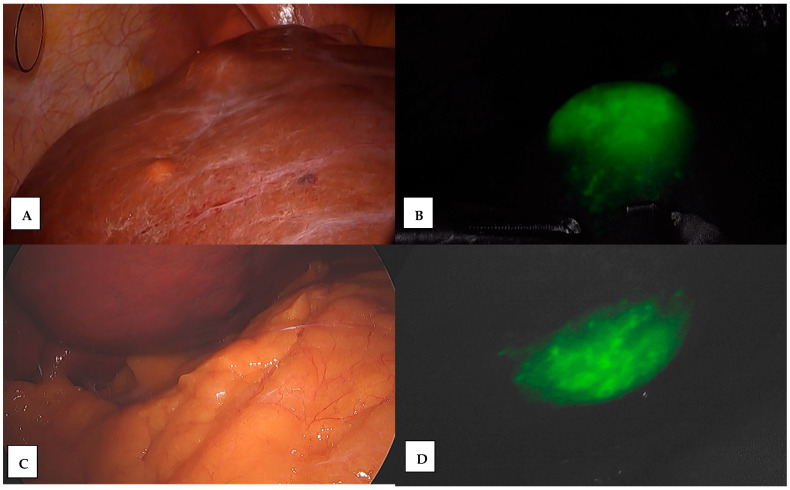
ICG use for liver tumor localization. (**A**) Tumor of superior surface of the VII hepatic segment, (**B**) ICG localization and delimitation, (**C**) tumor of inferior surface of the V hepatic segment, and (**D**) ICG localization and delimitation.

In the context of hepatobiliary surgery, ICG has also found applications in liver resection and transplant. According to a different study, ICG-navigated resection is useful for partial liver resection and anatomical liver resection for liver cancer and extended cholecystectomy for gallbladder cancer [64,65,66]. Recently, indocyanine green ICG imaging has been used to identify hepatic tumors and segmental boundaries during hepatectomy (Figure 4). Based on the review of Takemura N et al., the advantages of ICG fluorescent segmental staining are its high reproducibility and sensitivity [67]. It stays in the injected segment for a few hours because ICG is taken up by hepatocytes. Moreover, the segmental border inside the liver can also be visualized by this technique, thus providing intraoperative navigation. Regarding another unique application of ICG fluorescence imaging, Kawaguchi et al. applied ICG fluorescence imaging to investigate the decreased functional reserve of the venous congestion area after the resection or clumping of drainage veins [68]. They used the difference in fluorescence intensity, which was interrupted due to venous congestion and subsequent interrupted portal uptake function. The effect of the venous congestion area on liver function remains unclear. Sano et al. evaluated the venous congestion area in liver grafts for liver transplantation [69]. They reported portal flow regurgitation in the venous congested area and the necessity of venous reconstruction in liver grafts. However, the opening of peripheral venous communication released venous congestion in some patients. Mise et al. recommended subtracting the volume of the venous congestion area from the future functional remnant liver volume that is calculated before major hepatectomy [70]. Another use of ICG in liver transplant surgery is to assess if the donor’s liver is suitable before transplantation. By injecting ICG into the donor’s bloodstream, surgeons can evaluate the liver’s function and blood flow using ICG clearance parameters, helping them to determine whether the organ is suitable for transplantation. This preoperative assessment can help reduce the risk of complications and improve the overall success of the procedure. After the transplantation, ICG can be used to continue monitoring the graft’s function and blood flow during the postoperative period using ICG kinetics during the following days after transplantation; this includes the evaluation of ICG clearance just like in the preoperative phase. This can help detect any issues early on and guide the management of the patient’s care [71].

### 3.6. ICG in Bariatric Surgery

Bariatric surgery is currently a widely adopted therapeutic option for patients with morbid obesity. The advancements in laparoscopic techniques and instruments, coupled with recent refinements in procedure selection, have improved the safety and efficacy in the treatment of patients with obesity. However, this surgery, performed in non-neoplastic subjects, could lead to potentially fatal complications such as anastomotic leak, which not only increases morbidity and mortality but also results in additional recovery time, corrective procedures, increased healthcare costs, and potential malpractice. Currently, the incidence of total major adverse events at 30 days is 5.0% for gastric bypasses and 2.6% for sleeve resections, with the occurrence of leaks ranging from 0.4 to 2.7% of cases [72,73,74]. As reported by a systematic review by Hsu A. et al., ICG can be used intravascularly for vascular mapping and assessing aberrant arterial anatomy, and to assess blood supply of gastric sleeve or anastomoses during Roux and Y gastric bypass (RYGB) or biliopancreatic diversion [75]. Moreover, as discussed by Ortega et al., the identification of aberrant anatomy with ICG can lead to a change in surgical management to reduce the risk of complications. Especially with the complicated vascular anatomy of the GE junction and the variation in vascular patterns that patients can have, ICG becomes even more crucial in allowing for meticulous dissection that preserves the blood supply of the proximal stomach [76].

## 4. Acute Care and Trauma Surgery

**Figure 5:**
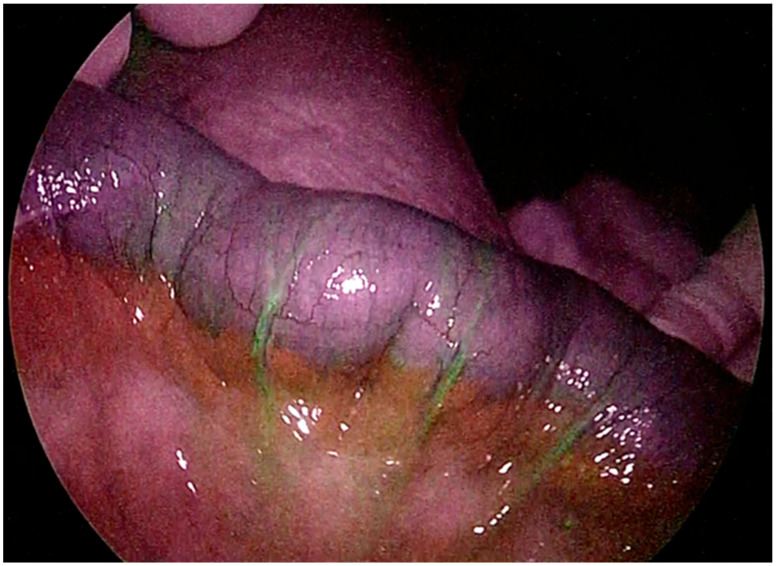
Evaluation of bowel during emergency laparoscopy for acute mesenteric ischemia.

Although there are many studies on the utility of ICG in elective surgery, to date, there is still a lack of literature regarding its use during emergency surgery, in which the surgeon and the patient can benefit the most from its use. In the acute care setting, ICG can be applied in different fields such as trauma, acute cholecystitis, and intestinal ischemia [77,78]. Overall, it seems that the use of ICG does not prolong surgical time in acute settings but does not decrease the need for damage control operations in an acute care surgery population. Based on our previous meta-analysis, the routine use of ICG fluorescence could modify intraoperative strategies and improve patient outcomes [79]. The benefits of using ICG fluorescence for acute cholecystitis, in terms of the clear identification of biliary structures, were reported in a significant percentage of patients [80,81,82,83,84]. For patients with mesenteric ischemia or trauma, the use of fluorescence resulted in a change in surgical approach in a notable percentage of cases with both minimally invasive and open approaches. Acute mesenteric ischemia remains a problematic issue with high mortality rates [85,86]. Emergency surgery, often with extended intestinal resections in order to remove the affected tissue, is necessary (Figure 5). Despite the high mortality rate associated with mesenteric ischemia, no real progress has been made in improving the survival of those patients in the last decade [87,88,89,90,91]. In the study by Ryu et al., the use of ICG in patients with strangulated bowel obstruction led to fewer resections and fewer complications [92]. Moreover, Karampinis et al., in a retrospective analysis of 52 patients with acute mesenteric ischemia who underwent surgery, concluded that the use of ICG fluorescence angiography is a possible and reliable tool for perfusion assessment of the bowel, with a rate of clinical benefit of 11% [93]. He also compared macroscopic inspection with ICG and reported a discrepancy rate of 35%, which is relevant for the correct identification of the resection margin [94,95,96,97]. Regarding trauma, studies on the use of ICG fluorescence are still limited and have not focused on a minimally invasive approach: ICG has been utilized to evaluate the viability of parenchymatous organs, assess tissue impairment related to perfusion in extremity or craniofacial traumas, and reevaluate the vascularization efficacy of surgical procedures. This improves the clinical outcomes of surgery and patient safety [19,98,99,100,101].

## 5. Thoracic Surgery

Indocyanine green finds various applications in thoracic surgery such as complete resection of pulmonary bullae, precise demarcation of the segmental plane, pulmonary nodule localization, and the tracing of sentinel lymph nodes (SLNs) [102,103]. Regarding pulmonary bullae, while pulmonary bulla resection via video-assisted thoracoscopic surgery is the preferred treatment for most patients with spontaneous pneumothorax, studies have indicated a higher recurrence rate with minimally invasive surgery, primarily attributed to residual bullae [104,105,106,107]. Compared to adjacent normal lung tissue, bullae or emphysema lesions exhibit lower tissue density and reduced blood flow, resulting in a lower concentration distribution of ICG following intravenous injection. Real-time near-infrared fluorescence imaging allows for the immediate visualization of a sharp decline in the ICG concentration, enabling clear differentiation between bullae and normal lung tissue. Matsumoto et al. employed ICG to accurately outline the diseased lesion using near-infrared fluorescence imaging in a patient with a sizable emphysematous lesion in the right lower lobe [108].

Positioning sub-centimeter pulmonary nodules accurately during video-assisted thoracoscopic surgery remains a considerable challenge. Traditional methods such as hook-wire insertion, spring coil placement, and methylene blue injection are associated with complications like hook-wire slippage, dye dispersion, pneumothorax, and air embolism [109,110]. Studies have indicated that due to the enhanced permeability and retention effect, ICG can accumulate in tumor tissue while sparing lung tissue and preserving pathological diagnostic accuracy [111,112,113,114,115]. Okusanya et al. administered intravenous ICG 24 h prior to surgery, resulting in a nodule detection rate. In recent years, indocyanine-green-guided near-infrared (ICG NIRF) imaging technology has also been applied clinically to the precise demarcation of the segmental plane [116]. Sekine et al. administered ICG directly into the target pulmonary segment via the trachea. They then utilized fluorescent thoracoscopy to visualize the segmental plane and precisely dissect the target segment [117]. Furthermore, Mun’s study demonstrated that intravenous systemic injection of ICG fluorescence yielded clear and sustained demarcation of the lung surface in a significant percentage of patients [118,119,120].

## 6. Urologic Surgery

Golijanin et al. investigated the mechanism underlying ICG fluorescence in the kidney, utilizing immunohistochemistry with polyclonal antibodies [121,122]. Their study revealed the presence of bilitranslocase, a membrane protein, in the proximal and distal convoluted tubules of normal kidney cells. Bilitranslocase facilitates the transport of organic anionic molecules like ICG from the blood into the cell. However, its down-regulation in renal cortical tumors results in decreased ICG uptake, explaining the hypofluorescence observed in cancerous cells under ICG near-infrared fluorescence compared to normal and benign tissue. The mechanism of intraureteral ICG use is less understood. Unlike kidney cells, urothelial cells of the urinary tract lack absorption capabilities. Nevertheless, intraureteral ICG injection allows for the identification of the ureter and differentiation between viable and nonviable ureteral tissue. One hypothesis suggests that ICG binds to urothelial surface proteins in viable ureteral tissue but not in nonviable tissue [25,123]. While ICG has not yet been approved by the FDA for intraurethral use, the technique shows great promise. In urologic surgery, ICG can play multiple roles [25,124,125,126,127]. Several authors report the role of ICG in tumor localization during partial nephrectomy [128,129,130]. In 2011, Tobis et al. published their initial clinical findings involving patients who underwent near-infrared fluorescence imaging with ICG during robotic partial nephrectomy. The objective was to utilize ICG to distinguish between normal and malignant tissue and visualize the renal vasculature. Among the patients, the majority were found to have malignancies upon final pathology examination [131]. In 2015, Bjurlin et al. conducted a literature review on ICG-guided tumor localization during robotic partial nephrectomy. They highlighted promising but inconsistent results due to variations in ICG dosing [132]. Angell et al. aimed to refine dosing strategies and achieve histologic prediction accuracy. While this accuracy was deemed insufficient for guiding tumor margin resections, the authors suggested it could benefit less experienced surgeons [133]. In Italy, researchers introduced a novel technique for the near-infrared fluorescence localization of renal tumors. They utilized preoperative renal angiography and super-selective transarterial delivery of a lipiodol–ICG mixture to improve the visualization of tumor margins in off-clamp partial nephrectomies. Although the initial results showed improved visualization and negative surgical margins for all patients, comparative data for this technique are currently lacking [134,135]. Moreover, during partial nephrectomy, ICG can be used as a guide for selective arterial clamping. Using near-infrared fluorescence with ICG selective arterial clamping offers surgeons an intraoperative renal angiogram. This enables them to selectively clamp minor arteries rather than the main renal artery. The objective is to enhance long-term functional outcomes by minimizing ischemia in normal renal parenchyma [136,137]. In 2012, Borofsky et al. conducted a matched-pair analysis comparing robotic partial nephrectomy outcomes using indocyanine green-guided selective arterial clamping vs. conventional main renal artery clamping, all performed by the same surgeon. The short-term follow-up revealed only a decrease in the estimated glomerular filtration rate in the selective arterial clamping group, in contrast to a decrease in eGFR observed in the conventional main renal artery clamping group [138]. Another promising application of ICG in urology is the identification of the lymphatic pathway in prostatic cancer. Prostate cancer ranks among the most prevalent urinary cancers in men worldwide [139,140,141]. Although prostate-specific membrane antigen (PSMA) imaging is the gold standard for lymph node staging in prostate cancer, its sensitivity varies according to lymph node size and Prostasti Specific Antigen (PSA) level. The limitations of PSMA imaging, including the invasiveness, high cost, and potential adverse effects of anesthesia, restrict its widespread application. Sentinel lymph node biopsy (SLNB) offers an alternative method for lymph node staging, with lower morbidity and comparable sensitivity. Despite extensive research, there remains no consensus regarding the optimal technique for SLNB in prostate cancer, with variations in the choice of tracer and injection site [142]. Another application of ICG in urologic surgery is during the Laparoscopic Palomo varicocelectomy. As reported by Esposito C. et al., this is a standardized technique to perform lymphatic sparing and avoid post-operative hydrocele. However, while no data regarding the safety of intratesticular injection of ICG are currently available, they reported the safety of intratesticular injection of ICG at mid-term follow-up, without specific risks for either the testis or the patient [143].

## 7. Gynecological Surgery

The clinical application of ICG in gynecologic surgery has garnered significant interest in recent years. Gynecologic oncology encompasses various malignant conditions, including cervical, endometrial, ovarian, vaginal, and vulvar cancers. Intraoperative ICG fluorescence imaging has emerged as a promising adjunct to conventional surgical techniques, offering real-time visualization and localization of sentinel lymph nodes (SLNs) and enhanced identification of tumors. The feasibility and accuracy of ICG fluorescence imaging for SLN mapping in gynecologic cancers were first demonstrated in studies investigating cervical cancer [144,145,146,147,148,149,150,151,152,153]. Cervical cancer commonly metastasizes via the lymphatic system, with pelvic and para-aortic lymph node involvement being key prognostic factors. SLN mapping aims to accurately identify and remove the initial lymph node(s) receiving lymphatic drainage from the primary tumor, potentially avoiding the need for extensive lymphadenectomy and reducing associated morbidity. Beyond cervical cancer, the application of ICG in other gynecological malignancies has shown promising results. In endometrial cancer, ICG has been utilized to enhance the detection of SLNs, aiding in precise staging and reducing the likelihood of overtreatment. Studies have also indicated the potential of ICG in improving the visualization of SLNs in patients with early-stage ovarian cancer, thereby aiding in the surgical staging process and potentially impacting treatment decisions. Moreover, the use of ICG fluorescence imaging has extended to minimally invasive surgical approaches, such as laparoscopy and robotic-assisted surgery. These techniques benefit from the enhanced visualization capabilities of ICG, allowing surgeons to perform more precise dissections and reduce the risk of complications. The integration of ICG fluorescence imaging into these advanced surgical modalities represents a significant advancement in the field of gynecological oncology. In addition to SLN mapping, ICG has been employed to delineate tumor margins more accurately during surgery, particularly in complex cases where distinguishing between malignant and healthy tissue is challenging. This capability is crucial for ensuring complete tumor resection while preserving as much healthy tissue as possible, ultimately improving patient outcomes. Minimally invasive surgery for cervical cancer may risk peritoneal contamination. Klapdor et al. [154] proposed using ICG to detect contamination during colpotomy. This approach, though based on a single study, could serve as a quality assessment tool for surgical techniques. Pelvic exenteration is the final curative option for patients with recurrent or persistent gynecological tumors who have previously undergone radiotherapy. This surgery is associated with high morbidity and complications related to urinary diversion. Intraoperative ICG can be used to assess ureteral perfusion. Studies have reported that better intraoperative ureteral perfusion, as assessed by ICG, correlates with reduced rates of benign ureteral stenosis. For instance, a pilot study by Bizzarri et al. [155] utilized intraoperative angiography with ICG to evaluate ureteral-ileal anastomoses, ileo-ileal anastomosis, and the ileal conduit, though the small number of patients prevented significant conclusions. Despite limited evidence, ICG angiography shows promise in reducing morbidity associated with pelvic exenteration, warranting further prospective studies to validate its efficacy. Moreover, in gynecological cancer surgeries, radical procedures often involve total hysterectomy followed by vaginal suture, which can be performed abdominally in open approaches or laparoscopically/vaginally in minimally invasive settings. A complication, vaginal cuff dehiscence (VCD), occurs at varying rates depending on the surgical method. Risk factors for VCD include premenopausal status, smoking, surgical-site infection, and chemotherapy. ICG administration can evaluate vaginal cuff vascularity during surgery. Studies by Beran et al. in 2016 [156,157] on laparoscopic and robotic hysterectomies validated ICG’s use for assessing vaginal cuff vascularization. Prospective trials are needed to confirm these findings and potentially reduce postoperative VCD rates through intraoperative suture revisions and closer follow-up for high-risk patients. Radical vulvectomy, the main treatment for vulvar cancer, sometimes necessitates flap reconstruction for large or recurrent tumors. ICG angiography, although limited to a few case reports, offers a simple and affordable method to verify flap vascularization. In a series by Capozzi et al. [158], patients undergoing radical vulvectomy with flap reconstruction had no surgical infections, dehiscence, or necrosis. ICG angiography for flap viability, described by Gentileschi et al., showed no further complications, suggesting its potential for quick intraoperative assessment. Technological improvements are necessary to enhance this method’s reliability. Moreover, ICG’s therapeutic potential in gynecological cancers includes photothermal and sonodynamic therapies, combined with chemotherapy. Nanocarriers encapsulating ICG enhance its stability and targeting ability. Preclinical models show promising results for a tailored therapeutic approach, but further research is necessary to advance these technologies. Overall, the incorporation of ICG fluorescence imaging into gynecological oncology surgery offers numerous benefits, including enhanced accuracy in SLN mapping, improved tumor localization, and better surgical outcomes. Ongoing research and technological advancements are likely to further expand its applications and efficacy in the management of gynecological cancers [144,145,146,147,148,149,150,151,152,153,154,155,156,157,158,159].

## 8. Pediatric Surgery

ICG technology offers significant advantages in pediatric surgery. Moreover, a review by Breuking EA et al. [160] reported that ICG may be safe to use in neonates since all but one study reported the absence of any complication or adverse event occurring when ICG was used in infants ≤ 3 months. Two studies reported side effects of retention of the injected dye or a change in skin color after the use of ICG, but these effects are temporary and fully disappear in several weeks. ICG has the same applications as in adults concerning lymph node mapping, the evaluation of anastomotic perfusion, and the assessment of the extent of intestinal resections [161]. Additionally, it has specific applications related to pediatric-specific conditions: in pediatric urology, ICG is used for assessing renal perfusion and visualizing the urinary tract for conditions like hydronephrosis, ureteropelvic junction obstruction, and kidney transplantation [162]. Another very good indication of ICG-enhanced fluorescence in pediatric surgery is intraoperative lymphography during laparoscopic varicocelectomy to separate the lymphatic vessels from the blood vessels, avoiding postoperative hydrocele formation [163].

In gastrointestinal surgeries, ICG can be used to assess bowel perfusion, particularly in cases of intestinal atresia, necrotizing enterocolitis, and other congenital anomalies. It helps ensure the viability of bowel segments before anastomosis. In congenital vascular malformations and pediatric vascular surgeries, ICG helps to assess the patency of blood vessels and the effectiveness of anastomoses. It is also valuable in evaluating tissue perfusion during reconstructive procedures [160]. Experience in pediatric surgery, however, remains limited, and the indications of ICG-guided NIRF are not yet as clear in pediatric patients.

## 9. Limitations

There are some pitfalls that have to be addressed: the accuracy of ICG fluorescence can be affected by various factors leading to false-positive or false-negative results. For instance, inflammation, scarring, or other pathological changes can alter the uptake and distribution of ICG, potentially leading to misinterpretation. Moreover, there is a lack of standardized protocols for the use of ICG fluorescence. Variations in the dosage, timing of administration, and imaging techniques can lead to inconsistent results across different practitioners and institutions. Lastly, the equipment required for ICG fluorescence imaging, including near-infrared cameras and specialized software, can be expensive and may not be readily available in all surgical centers, particularly in low-resource settings.

## 10. Conclusions

IGC fluorescence imaging represents a versatile tool in various surgical specialties, including general surgery, acute care surgery, thoracic surgery, urologic surgery, pediatric surgery, and gynecological surgery. Its real-time visualization capabilities enable surgeons to improve precision and safety during complex procedures, ranging from tumor localization and lymph node mapping to perfusion assessment and tissue viability evaluation. Despite ongoing research efforts, further investigation is warranted to elucidate optimal dosing regimens, injection techniques, and imaging protocols, with the ultimate goal of optimizing patient outcomes and enhancing the standard of care in surgery.

## Figures and Tables

**Table 1:** Summary of indocyanine green usage in surgery.

Disease	Objective	Method of Injection	Timing	Dose
Cholecystectomy	Anatomy identification/Bile duct visualization	I.V./intra-gallbladder	At least 45 min before procedure/during surgery	0.4 mL 2.5 mL, equipment dependent
Hemicolectomy	Perfusion assessment/extension of resection	I.V.	Intraoperatively	3–3.5 mL + 10 cc saline flush
ureter identification	Visualization of ureters	Cystoscopic-guided retrograde intraureteral ICG	Prior to pelvic dissection	2.5 mg/mL 2 mL per ureter
Esophagectomy	Gastric conduit perfusion evaluation	I.V.	Intraoperatively	3 mL + 10 cc saline flush
Lymph node mapping	Sentinel lymph node localization	Peritumoral area	Preoperatively or intraoperatively	0.5–1 mL on each tumor quadrant
Liver resection	Perfusion assessment/Direct identification and resection	I.V./Positive staining technique: inject in portal branch	Prior to hepatic dissection	2.5 mg per body/0.25–2.5 mg/10 mL
Liver transplant	Liver function and blood flow	I.V.	From 2 h to 20 min before surgery/during surgery	0.4 mL 2.5 mL, equipment dependent
Bariatric surgery	Perfusion assessment	I.V.	From 2 h to 20 min before surgery/during surgery	0.4 mL 2.5 mL, equipment dependent
Pulmonary bulla resection/Pulmonary nodules	Direct identification and resection	I.V.	during surgery	0.4 mL 2.5 mL, equipment dependent
Partial nephrectomy	Direct identification and resection	I.V.	during surgery	0.4 mL 2.5 mL, equipment dependent
Laparoscopic Palomo varicocelectomy	Lymphatic sparing	intratesticular	during surgery	0.4 mL 2.5 mL, equipment dependent
